# Lectures on the Theory and Practice of Midwifery, Delivered in the Theatre of St. George’s Hospital

**Published:** 1844-07-27

**Authors:** 


*Lectures on the Theory and Practice of Midwifery, delivered in the Theatre of St. George's Hospital.* By Robert Lee, M. D., F. R. S., &c. &c. *Illustrated with numerous wood engravings.* Philadelphia: Ed. Barrington & Geo. D. Haswell, 1844. 8vo. pp. 540.

Here is another valuable book for which the country is in debt to the Select Medical Library, too well known by its Editor Dr. Bell to require praise from us. We doubt if the Sydenham Society of London, aided by the universal contribution of the profession, will be able to exhibit a more commendable activity, or a more judicious discrimination in the publications we are to hope for under its auspices, than are constantly manifested by the manager of the Select Medical Library—which not only caters for us in all the *nouveautés* but serves us up such solid and useful materials as the present, and the great variety of other publications of great merit, which shine on our shelves—thanks to its liberality. But let us not be diverted from our purpose, which is to say a few words about Dr. Lee's volume. This work consists of 44 Lectures delivered as above by Dr. Lee, at St. George's, and reported in the London Medical Gazette for 1842-3. They have been revised by Dr. Lee himself, and reprinted in a volume. For the American copy before us we are indebted to Dr. John Bell.

Dr. Lee has bestowed considerable pains upon the physiological part of the subject, and in particular has he entered into a discussion of the new doctrines of menstruation, which really taking their rise under the auspices of M. Negrier, at the same time that they attracted the regard of M. Gendrin, have made rapid progress towards a complete and firm establishment by means of Lee, Raciborski and others. It is known that this doctrine professes that menstruation depends upon a periodical excitation of the ovaries and uterus, proceeding from a normal, regular production of Graafian vesicles, which are supposed to reach the surface of the ovary when mature, and then to burst for the discharge of the ovule contained within, and that one such vesicle is developed in each month. The growth of the vesicle, which is rapid at last, cannot but press aside the ovarian stroma, and irritate the ovary, which becomes the seat of an hyperæmic fluxion that involves the whole womb, and in some cases a part of the vagina. This hyperæmia is relieved by the menstrual hæmorrhage, or courses. To show what Dr. Lee thinks on this point, we extract from the top of page 46, the following:

"That the determination of blood which takes place to the uterine system every month, and that all the phenomena of menstruation depend upon the ovaria, and that at each period a Graafian vesicle bursts, and its contents escape, is rendered extremely probable by the following facts."

The facts cited are, the absence of menstruation in such as are congenitally or accidentally deprived of ovaries, and the cessation of menstruation in cases where the ovaries are destroyed by disease. He states that women without womb, but possessing ovaries, have all the periodical symptoms of catamenial power, except the flow. Dr. Lee recalls, at p. 47, the history of an autopsy performed by him on the 11th of March, 1831. It was on the body of a woman who died while menstruating. She died with inflammation of the median basilic vein. In this case Dr. L. found the pit on the ovary, the locus (in quo) of the ruptured vesicle, the ovary. The Fallopian tubes, and the womb, were all red, and the tubes and uterus contained what was supposed, very correctly, to be menstrual fluid. Dr. Lee speaks of other cases, and cites those to be found in Gendrin, (Traité de Méd. Prat.) A warm controversy, says he, has been carried on between M. Negrier and Dr. Gendrin on the priority of the discovery, and they appear to have been wholly unaware, and which adds much to the importance of their observations, that an account of precisely the same appearances had not only been published by me seven years before, in the Cyclopædia of Practical Medicine, but fifty-eight years before, in the Philos. Trans. by Mr. Cruikshank—he also refers to the Kerckring's idea of a somewhat similar kind.

Now we have read the reclamations of M. N. Negrier and Gendrin—and we rest under a painful conviction that full justice is not done to M. Negrier, either by Lee, Gendrin or Raciborski. Columbus is the discoverer of America. This is true, though it is equally true that the Norsemen had repeatedly visited the shores of New England long before Columbus brought to light the truth of the theory of a western continent; and notwithstanding that Kerckring and Cruikshank and others may have had vague notions as to the menstrual development of the vesicle of De Graaf, we are indebted to M. Negrier for a clear exposé and elucidation, and, we may venture to add, firm establishment, of the theory of Menstruation. M. N.'s brochure, with his excellent drawings and accurately described cases, does him great credit, and we are so great lovers of justice that we would crown M. Negrier for establishing a most important point in physiology—a point on which turns such an immense variety and detail of pathology and therapeutics—palmam qui meruit ferat. We sincerely believe that M. Negrier has connected his name indissolubly with a most important discovery in physiology; and that whoever writes the History of Medicine in the nineteenth century, will be compelled to do him justice.

In this journal we cannot undertake to make reviews of medical works. Dr. Lee is too good and sensible an author to be reviewed. He should be read. He has already, though but a young man, become illustrious by his earlier works. He has cast a brilliant light upon the subject of the post puerperal maladies, as Crural Phlebitis, Uterine Phlebitis, and Peritonitis; and his charming little duodecimo—Clinical Midwifery—a copy of which he was so kind as to send us, is as entertaining as a romance, by the spirit and vigour with which he sketches circumstances that must ever be deemed fit to excite the warmest of human sympathies and interests. Of Dr. Lee we think it may be said, *nil tangit quod non ornat;* and those of our brethren who love to read sensible books by sensible men, will take pleasure and profit in the Lectures of this admirable physician. M.

